# Study on the Depth, Rate, Shape, and Strength of Pulse with Cardiovascular Simulator

**DOI:** 10.1155/2017/2867191

**Published:** 2017-01-29

**Authors:** Ju-Yeon Lee, Min Jang, Sang-Hoon Shin

**Affiliations:** ^1^Department of Cardiovascular Engineering, Institute of Applied Medical Engineering, Helmholtz Institute Aachen, RWTH Aachen University, Aachen, Germany; ^2^Department of Eastern-Western Biomedical Engineering, Sangji University, Wonju, Gangwon, Republic of Korea; ^3^Department of Oriental Biomedical Engineering, Sangji University, Wonju, Gangwon, Republic of Korea

## Abstract

Pulse diagnosis is important in oriental medicine. The purpose of this study is explaining the mechanisms of pulse with a cardiovascular simulator. The simulator is comprised of the pulse generating part, the vessel part, and the measurement part. The pulse generating part was composed of motor, slider-crank mechanism, and piston pump. The vessel part, which was composed with the aorta and a radial artery, was fabricated with silicon to implement pulse wave propagation. The pulse parameters, such as the depth, rate, shape, and strength, were simulated. With changing the mean pressure, the floating pulse and the sunken pulse were generated. The change of heart rate generated the slow pulse and the rapid pulse. The control of the superposition time of the reflected wave generated the string-like pulse and the slippery pulse. With changing the pulse pressure, the vacuous pulse and the replete pulse were generated. The generated pulses showed good agreements with the typical pulses.

## 1. Introduction

Pulse diagnosis is one of the most important diagnostic methods in oriental medicine. Previous studies on the modernization of pulse diagnosis can be divided into two groups, namely, the objectification of pulse measurements and the explanation of the pulse mechanism with science. This study focused on the explaining of the pulse parameters such as depth, rate, shape, and strength that are clinically relevant [1] to hemodynamics.

The depth of pulse is the depth of maximum pulse feeling with fingers. Fei [2] suggested the P-H (applied pressure-pulse amplitude) curve based on the clinical measurements by a pulse-taking device. According to the P-H curve, a floating pulse has maximum pulse amplitude at low applied pressure. In contrast, a sunken pulse has maximum pulse amplitude at high applied pressure. Various algorithms were suggested to distinguish between the floating pulse and the sunken pulse by measuring the changes of pulse amplitude based on the applied pressure or depth [3–5]. Fei [2] suggested the mechanism of the floating pulse and the sunken pulse with the blood pressure. The amplitude of pulse reached a maximum when the intravascular pressure and externally applied pressure were equal, and the P-H curve moved to the right as the inner pressure of the blood pressure increased. The pulse rate is the heart rate. The pulse shape is related to the width, the length, and the waveform of the pulse. Jang suggested that the large pulse and fine pulse were related to the blood flow [2]. Study on the length is rare. Shin et al. [6] indicated that the string-like pulse and the slippery pulse were related to the stiffness of blood vessel. Murgo et al. suggested that the pulse shape was determined by the superposition of the forward pulse wave and the reflected wave [7]. The pulse strength is the resistance of a blood vessel on a finger. In order to explain the vacuous pulse and the replete pulse, Fei suggested concepts like “pulse force” representing the magnitude of H in P-H curve and “pulse power” representing the area of P-H curve [7]. The concepts of pulse force [8, 9] and pulse power [10, 11] were tested with the clinical trials to distinguish between the vacuous pulse and the replete pulse, and the prediction accuracy by pulse force was higher. The dominant methods for previous studies were clinical trials that compared the results of pulse-taking device with those of doctors. This largely contributed to the quantification of pulse types. However, there are many limitations in explaining the mechanism of pulse, because it is impossible to independently control the influence factors for pulse.

There were a few studies that examined the pulse mechanism by simulation [12, 13]. The aging effect on the radial pulse wave was simulated with hemodynamic analysis [12]. The mechanisms of pulse depth were simulated with elastic string model in which the skin was modeled with the elastic string, the palpation pressure with tension in the string, and the blood pressure with external force on the string [13]. They were meaningful because analytical models were used to explain the mechanism of pulse. However, they had a restricted application area because of the assumptions in the model. Lee et al. [14] developed a cardiovascular simulator with an elastic arterial tree that could simulate the physical phenomenon of the arterial system. However, there were no studies that examined the mechanism of pulse with simulator. The purpose of this study is explaining parameters of pulse with a cardiovascular simulator.

## 2. Cardiovascular Simulator

### 2.1. Components of a Cardiovascular Simulator

**Figure 1:**
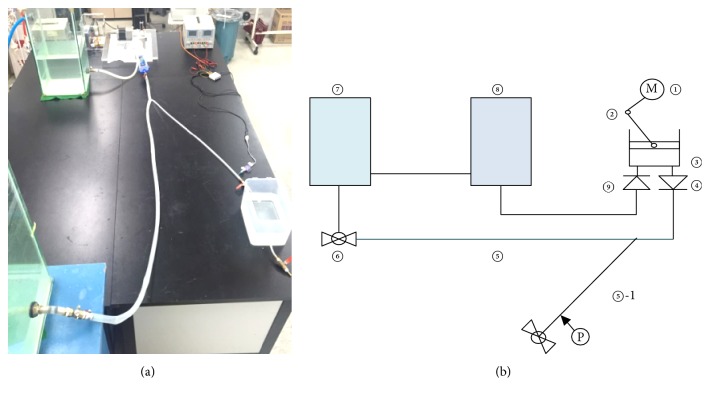
Cardiovascular simulator. (a) The simulator. (b) Diagram of simulator.

Figure 1(a) shows the developed cardiovascular simulator.  Figure 1(b) illustrates the composition of the simulator. The system is comprised of three parts, namely, the heart part, the vascular part, and the blood part. The simulator was configured with two reservoirs (peripheral resistance and the venous system: ⑦, left atrium: ⑧), two check valves (aortic valve: ④, atrioventricular valve: ⑨), pulse generating part (left ventricle: ①, ②, and ③), vascular system (⑤, ⑤-1), and resistance control valve (peripheral resistance: ⑥). The height of reservoir 1 (⑦) could be changed, and the resistance was accurately controlled by valve (⑥) and hydrostatic pressure (⑦). The rotational motion of the motor (①) was converted into a linear motion of the piston through a slider-crank mechanism (②) [15]. The working fluid flowing from reservoir 2 (⑧) into the cylinder via check valve 2 (⑨) was converted into a pulsatile flow by the linear motion of the piston and was then spurted out into the blood vessel part (⑤) via check valve 1 (④). The working fluid that passed through the blood vessel part flowed into the flow control valve (⑥), then into reservoir 1 (⑦), and finally back to reservoir 2 (⑧).

### 2.2. Heart Part

The heart part generates a pulsatile flow and is comprised of reservoir 2, check valves (④, ⑨), and a pulse generating part (①, ②, ③). The designed linear motion of the piston was converted into a rotational motion of the motor by computer aided inverse kinematic analysis [15]. The AC stepping motor with a maximum torque of 20 N was used. The check valve was designed to get activated at a small pressure difference [16].

### 2.3. Vascular System

In this study, a simplified arterial tree model, comprised of the aorta and the radial artery, was manufactured, as shown in Figure 1. With respect to the thickness and the inner diameter of the vascular part, dimensions of the aorta and the brachial artery were used to compensate the flow rate distribution for the brachial artery from the aorta.

The elastic modulus of the silicon tube used in this study was higher than that of the human body. So the generated pulse wave velocity was faster than that of the human body. The pulse wave velocity is the main factor for pulse wave formation because it determines the superposition time of the forwarding wave with the reflected wave [16]. The superposition time was controlled by varying the length of the silicon tube. The superposition time of the reflected wave is the product of the reflection distance and the inverse of pulse wave velocity [17]. The pulse wave velocity of the mock aorta tube used for the experiment was approximately 18 m/s, which was approximately 4 times that of the pulse wave velocity of an actual aorta (5 m/s) [18]. Therefore, the length of the mock aorta was determined as 4 times the length of the actual aorta (0.5 m) [19]. The pulse wave velocity of the mock radial artery tube used in the experiment was approximately 21 m/s, which was twice that of the pulse wave velocity of the actual radial artery and the brachial artery (10 m/s) [20]. Therefore, the length was determined as 1 m, which was twice that of the combined lengths of the brachial artery and the radial artery of the human body (50 cm–60 cm) [21].

### 2.4. Working Fluid

The hemodynamic characteristics are important for replicating blood. These are the density and the viscosity. A mixture of water and glycerin is used as a working fluid. In this study, the working fluid was composed of 37% glycerin and 63% water [22]. The density and the viscosity of blood were 1.06 g/cm^3^ and 3.5 cP, respectively, and those of the working fluid were 1.09 g/cm^3^ [23] and 3.2 cP [24], respectively, at 20°C. This shows that the working fluid used in this study has hemodynamic characteristics similar to those of blood. The glycerin-water solution used in this study was a Newtonian fluid whereas blood is a non-Newtonian fluid. According to a previous study [25], the flow in the femoral artery behaves like a Newtonian fluid. The diameter of the tube used in this study is larger than that of the femoral artery. Thus, we can assume that the blood behaves like a Newtonian fluid in this case. Therefore, replicating blood with a working fluid is reasonable [26].

### 2.5. Measurement System

The amplitude of the pulse was measured at the radial artery (*Ⓟ*) in Figure 1(b), which corresponded to the area of pulse diagnosis. An invasive blood pressure sensor (1620 Pressure Sensor, MSI Sensors, Inc.) was used to measure the internal pressure of blood vessels, and the amplitude of the pulse was measured by the measurement system developed in this study.

**Figure 2:**
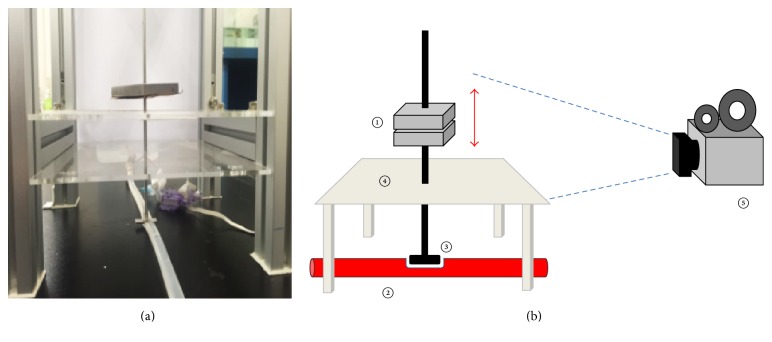
The pulse amplitude measurement system. (a) The system. (b) Diagram of the system.

Figure 2 shows the pulse amplitude measurement system used in the study. The following process was adopted to measure the amplitude of the pulse. The weights (①) were placed on the mock vessel (②) in order to apply pressure. The pressing device (③) was fixed by the holding device (④) and designed to press the vessel in the vertical direction. When the simulator was activated, the position of the moving weights was recorded with a fixed webcam (⑤: Logitech HD webcam c525) and saved in a personal computer. The unit of weight used for the simulation was 60 g and the applying pressure was increased with eight steps. The average contact area between the pressing device (③) and the vessel was approximately 0.88 cm^2^, and the increase of applied pressure for each step was approximately 50 mmHg. Therefore, the range of applied pressure was 50 mmHg–400 mmHg.

## 3. Method

### 3.1. Normal Pulse

**Figure 3:**
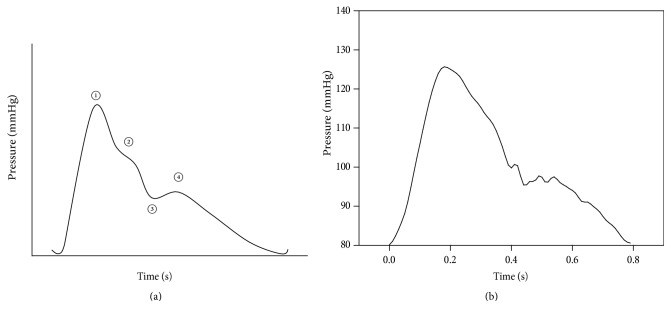
The normal pulse. (a) Typical waveform. (b) Generated waveform by the simulator.

The normal pulse is the pulse for a healthy adult and was used for comparison with other pulses [27]. A typical pulse wave is shown in Figure 3(a). In Figure 3, ① is the shock of left ventricular contraction, ② is the reflected wave, ③ is the incisura by the sudden closing of aortic valve, and ④ was created by the closure of aortic valve with consequent rebound of blood [27]. To simulate the normal pulse, a heart rate of 75 bpm [28] and a stroke volume of 60 mL [28] were used. The motor was rotated with a constant speed.  Figure 3(b) shows a generated normal pulse.

### 3.2. Depth

There are two points of view on the depth of pulse. The first one is the view on the vertical position of pulse feeling. The second one is the view on the response of pulse to the applied force on radial artery. With the tonometric measurement point of view, Lee et al. [29] suggested that the skin on the radial artery is the main factor in the former, while the mean pressure is the main factor at the latter. In the clinical test [30] with 12 oriental medical doctors and 169 subjects, the main factors for the depth of pulse were tissue thickness on the radial artery and the mean blood pressure. In this study, the depth of pulse was controlled by the mean pressure.

As the mean blood pressure was the product of the mean blood flow and the peripheral arterial resistance. Therefore the mean pressure was controlled by the peripheral resistance part (⑥, ⑦) in Figure 1(b). All other variables except the peripheral resistance were fixed. The coefficients of the floating and sunken pulses along with the P-H curve were obtained to determine the degrees of the floating and sunken pulses [4]. The coefficients of floating and sunken pulse (CFS) were as follows:

(1) $$\begin{array}{c} CFS=\left(\;\frac{Y_{1}+Y_{2}}{2}\;\right)-\left(\;\frac{Y_{4}+Y_{5}}{2}\;\right). \end{array}$$

In this equation, *Y*_1_, *Y*_2_, *Y*_4_, and *Y*_5_ corresponded to the pulse amplitudes of step 1, step 2, step 4, and step 5, respectively. The pulse was determined to be floating if the CFS was greater than *α* and sunken if the CFS was smaller than -*α*, where the constant *α* was determined by researcher.

### 3.3. Rate

The heart rate was controlled by the rpm of motor (①) in Figure 1(b), which was varied from 60 to 90 rpm. All the variables, except for the motor rpm, were fixed. The standards for the rapid pulse and slow pulse were just the number of pulses. According to the previous study [31], the bpm of the slow pulse was set to 60 and that of rapid pulse was set to 90 in this study.

### 3.4. Shape

The shape of pulse was controlled by the superposition time. The superposition time was affected by both the pulse wave velocity and travel length [17]. In this study, the travel length was changed to control the superposition time. In this study, the main reflection point of the pulse wave was at the end of aorta. Therefore, the length of aorta (⑤) in Figure 1(b) was changed in this study. The changed lengths were 2.4 m, 2.0 m, and 1.6 m. The length of 2.4 m represented the case with long travel length, and the length of 1.6 m corresponded to the case with short travel length. All the variables, except the length of aorta, were fixed.

### 3.5. Strength

The stroke volume of heart and the stiffness of blood vessel controlled the blood pressure [17]. In this study, the crank length (②) was changed to control the stroke volume. The peripheral resistance part (⑥, ⑦) in Figure 1(b) was also changed to generate the different pulse pressure values with the same mean pressure.

## 4. Results and Discussion

### 4.1. Depth

**Figure 4:**
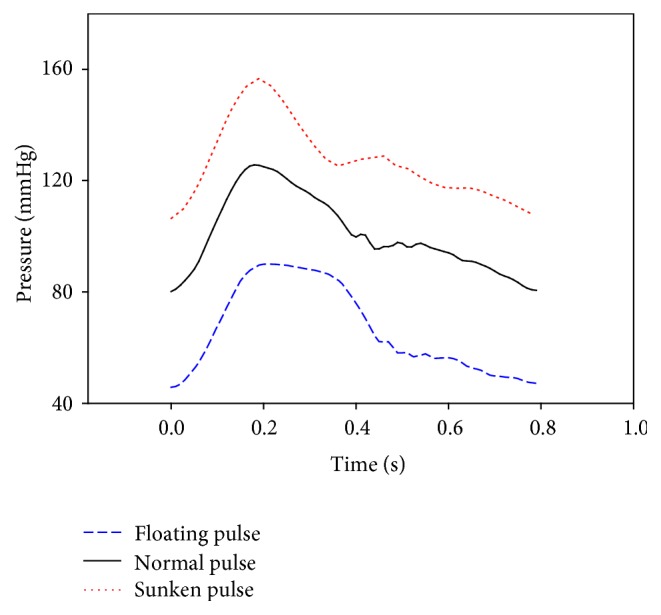
Simulated pressure waves of the floating pulse, normal pulse, and sunken pulse.

**Figure 5:**
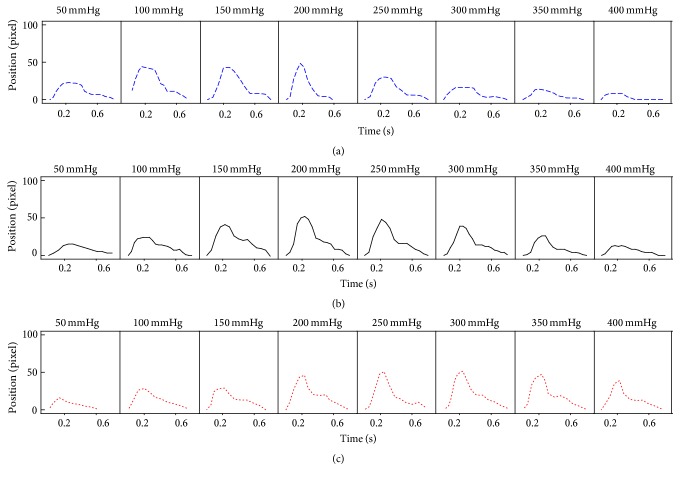
The measured pulse with increasing applying pressure for (a) floating pulse, (b) normal pulse, and (c) sunken pulse.

**Figure 6:**
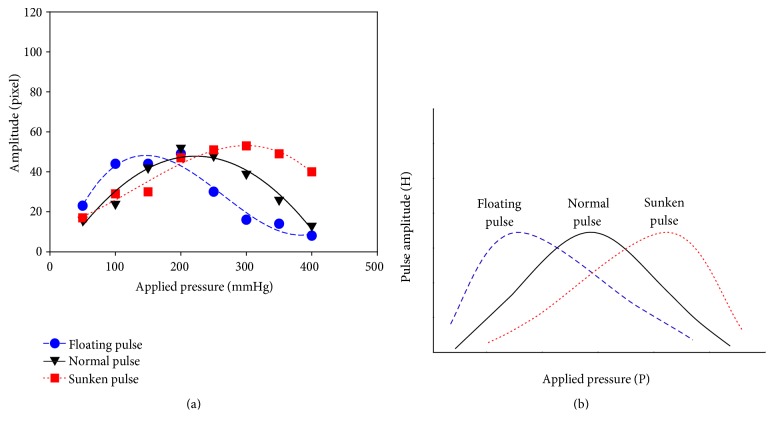
The P-H curve of floating pulse, normal pulse, and sunken pulse. (a) Measured P-H curve in simulator. (b) Typical P-H curve by Fei.

Figure 4 shows the pressure waves of the floating pulse and sunken pulse generated by the simulator.  Figure 5 shows the measured pulses with increasing applying pressure.  Figure 6(a) shows the P-H curve plotted from the results of Figure 5.  Figure 6(b) shows the typical P-H curve by Fei [2]. With the results of Figure 6(a), the coefficient of floating and sunken pulse was calculated. The CFS of the floating pulse, the normal pulse, and the sunken pulse was 23, 2, and −17.5, respectively

Figure 6(a) shows the results of P-H curves for the floating pulse, the normal pulse, and the sunken pulse. The peak of the P-H curve is located on the left (low applied pressure) for the floating pulse and on the right (high applied pressure) for the sunken pulse. The maximum amplitude of the pulse wave was similar to the three curves. In Figure 6(b), only the horizontal locations of the peak were changed, while there was no change in the maximum H value.  Figure 6(a) showed the same tendency. Therefore, it was experimentally confirmed that the mean blood pressure was the effective factor in the depth of pulse. The degree of floating and sunken pulse was calculated with the coefficient of floating and sunken pulse. According to the previous study [4], the CFS of floating pulse was higher than 15 and that of sunken pulse was lower than −15. As the CFS of generated pulses were 23, 2, and −17.5, they were in agreement with the suggested quantitative definitions.

### 4.2. Rate

**Figure 7:**
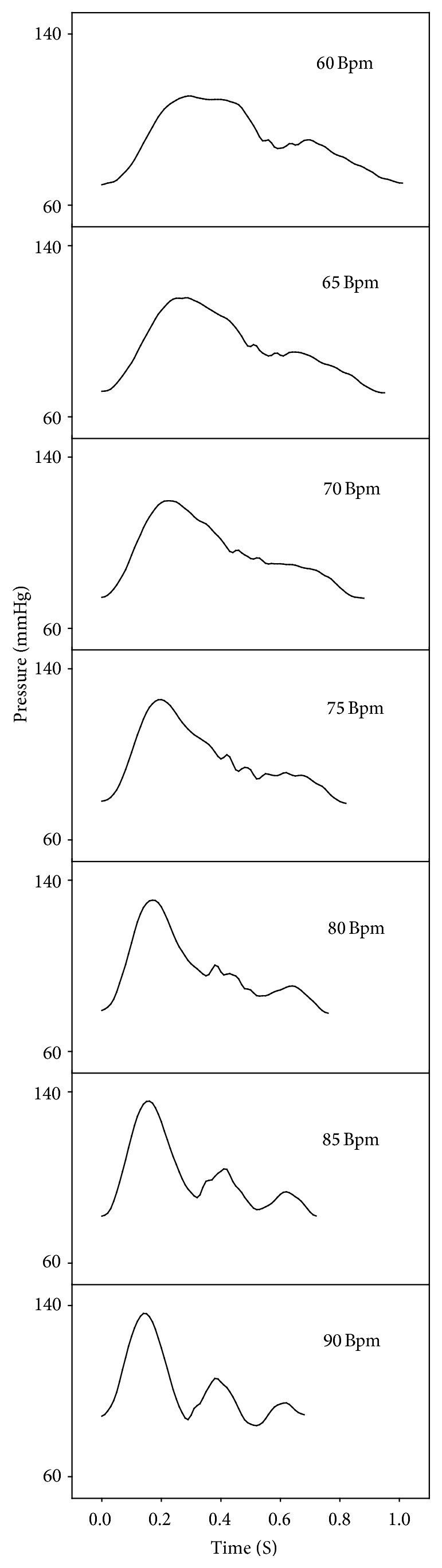
Pressure waves of the radial artery generated with various motor rpm.

The pulse rate is the heart rate.  Figure 7 shows the generated pulses with various heart rate. The change of heart rate means the change of pulse period. The pulse wave velocity remains constant in spite of the change of pulse period. In the pulse period, the timing of superposition with the reflected wave was changed. The superposition timing of the slow pulse was in the middle of the systolic period, while that of the rapid pulse was in the diastolic period. Therefore, the heart rate changed the waveform of pulse.

### 4.3. Shape

**Figure 8:**
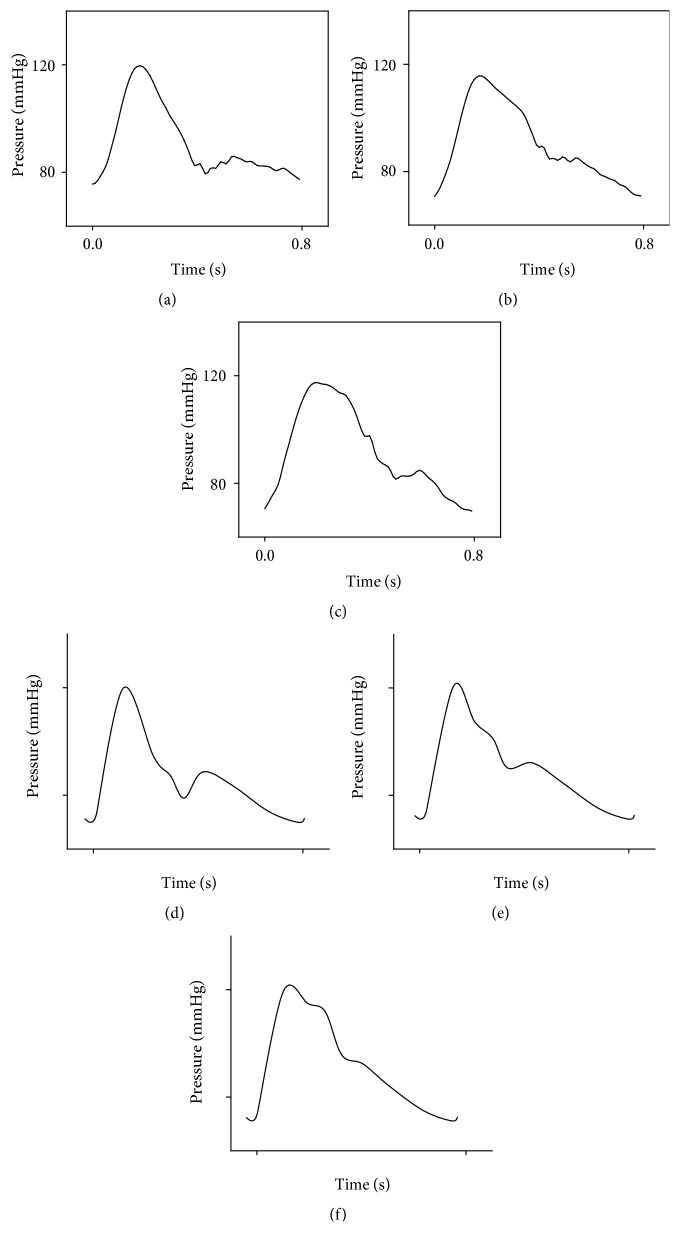
The pressure waves of radial artery generated with various aortic lengths which are (a) 2.4 m, (b) 2.0 m, and (c) 1.6 m. The typical waveforms of (d) the slippery pulse, (e) the normal pulse, and (f) the string-like pulse.

Figure 8 shows the pulse waves generated with the various aortic length. The pulse types related to the shape of the pulse wave were the string-like pulse and the slippery pulse. The characteristics of the pulses were compared with the normal pulse in Figure 8(e). The slippery pulse in Figure 8(d) showed rapidly increasing and decreasing waveform during the systolic period, had a noticeable waveform in the diastolic period, and had a low incisura pressure [6, 32]. The string-like pulse in Figure 8(f) has a superposition timing in the early systolic period and a high incisura pressure [6, 32]. The generated slippery pulse in Figure 8(a) and the string-like pulse in Figure 8(c) showed good agreement with the typical ones.

Murgo et al. [7] reported three pressure wave patterns of the ascending aorta in young and old subjects. They interpreted these three patterns as superposition of the reflected wave. The pulse wave velocity is increased with aging [6]. Figure 8 showed the effects of superposition time on the pressure wave of radial artery. In this study, the diameter and thickness of silicon tube were not changed, so the pulse wave velocity was constant. The superposition time was controlled by varying the length of the silicon tube in this study. In order to delay the superposition time, the length of silicon tube was increased. In case of the delayed superposition time, the slippery pulse was measured at the radial artery. In case of the shortened superposition time, the string-like pulse was measured at the radial artery. In this study, the shapes of pulse were simulated by controlling the superposition time of the reflected wave. It was experimentally confirmed that the mechanisms of the string-like pulse and of the slippery pulse were related to the superposition time of the reflected wave.

### 4.4. Strength

**Figure 9:**
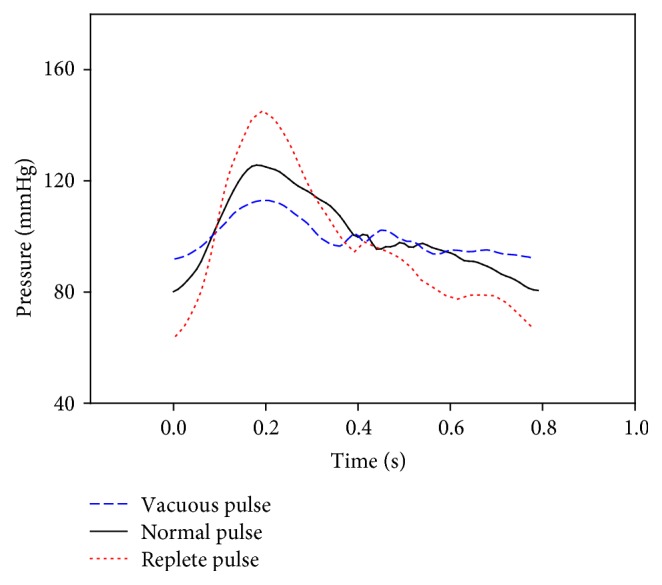
Simulated pressure waves of the vacuous pulse, normal pulse, and replete pulse.

**Figure 10:**
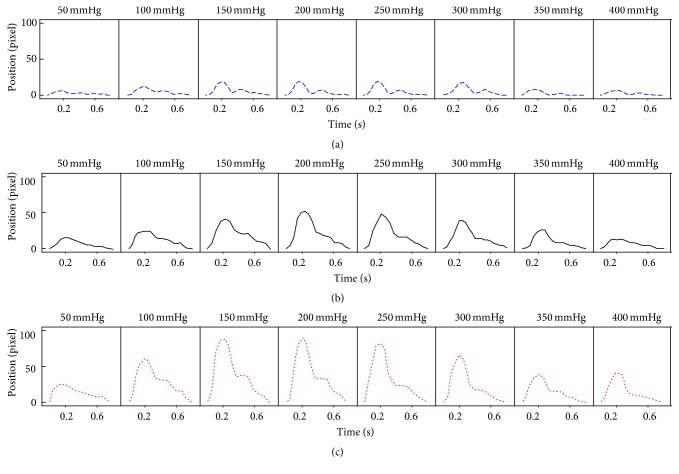
The measured pulse with increasing applying pressure for (a) vacuous pulse, (b) normal pulse, and (c) replete pulse.

**Figure 11:**
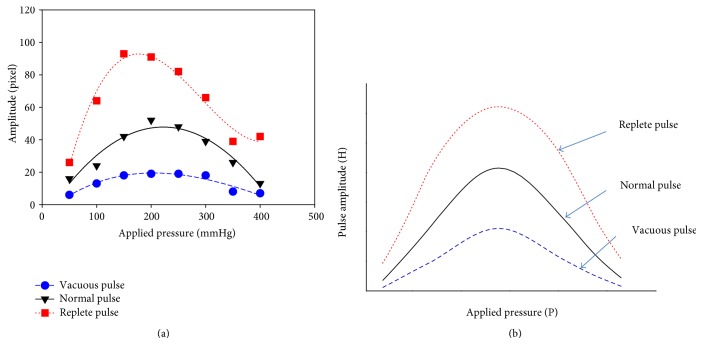
The P-H curve of vacuous pulse, normal pulse, and replete pulse. (a) Measured P-H curve in simulator. (b) Typical P-H curve by Fei.

Figure 9 shows the generated vacuous pulse and replete pulse.  Figure 10 shows the measured pulse with increasing applying pressure for the vacuous pulse and the replete pulse.  Figure 11(a) shows the P-H curve plotted from the results of Figure 10.

Figure 11(b) shows the typical P-H curves of the vacuous and the replete pulse [2]. In the P-H curve of Figure 11(a), only the heights of H were changed with the peak located in the center and not skewed to the right or left. This showed the good agreement with the typical P-H curve in Figure 11(b). Therefore, it was experimentally confirmed that the strength of the pulse was related to the pulse pressure.

This study had a few limitations. The camera used for recording was suitable for measuring the amplitude of the pulse but it did not provide a smooth pulse waveform. A more in-depth study would be possible with a more high resolution camera.

## 5. Conclusion

In this study, the pulse parameters such as the depth, rate, shape, and strength were simulated with a cardiovascular simulator. The depth of pulse was simulated by changing the mean pressure with constant pulse pressure. The strength of pulse was simulated by changing the pulse pressure with constant mean pressure. The rate of pulse was simulated by changing the rpm of motor. The shape of pulse was simulated by changing the superposition time of the reflected wave. The generated pulses showed good agreements with the typical pulses.
